# A Never Described Variant of the Cervical Rib Causing Arterial Thoracic Outlet Syndrome: World's First Case

**DOI:** 10.1055/s-0041-1731654

**Published:** 2021-07-22

**Authors:** Saif Abdeali A. Kaderi, Pravin Shinde, Raviraj Tilloo, Sonewane Chetan, Tanvi Dalal, Sahil Vaghmare, Dhaval Bhesaniya, Sulay Shah, Sameer Rege

**Affiliations:** 1 Department of General Surgery, Seth GSMC and KEMH, Mumbai, Maharashtra, India

**Keywords:** cervical rib, subclavian artery thrombosis, transverse process of the sixth cervical vertebra

## Abstract

Cervical ribs, also known as Eve's ribs, are rare and found in 1% of population. They are more common in females and more common on right side. They are asymptomatic in 90% of cases. Cervical rib fused with transverse process of sixth vertebra is rarer. We present a case of dry gangrene of lateral three fingers with right radial and subclavian artery thrombosis with rest pain, due to right cervical rib fused with transverse process of sixth vertebra. After development of line of demarcation of the dry gangrene, patient was operated for excision of cervical rib and sixth cervical vertebral transverse process followed by Ray's amputation of right second finger. Postoperative course was uneventful. Patient was discharged with oral anticoagulation and a healthy wound in right hand.

Cervical rib is described as an anomalous, supernumerary, extra, or additional rib that arises from the seventh cervical vertebra due to elongation of its anterior (costal) element of its transverse process. Also known as “Eve's rib,” 1 the presence of a cervical rib was already mentioned in the works of Galen in the second century AD, and in sixteenth century by Vesalius. 2 Subsequently, it was classified into four major types based on length and union with the first rib in nineteenth century. 3 Lapayowker in 1960 first 4 identified a rare case, which described the elongation of the transverse process of the sixth cervical vertebra forming the cervical rib. A cervical rib could also originate from the sixth or the fifth cervical vertebrae, as per Tubbs et al. 5 In very rare instances, it can also originate from the fourth cervical vertebra. 6 A total of three case reports in the literature describe another rare variant having articulation of anterior tubercles of the transverse processes of both the fifth and sixth cervical vertebrae on the first rib. 7 8 However, this anatomical variant was described to be asymptomatic as it did not cause any significant clinical dysfunction. Arteriothromboembolic events due to cervical rib compression of the subclavian artery are rare. Pressure of the cervical rib upon the right subclavian artery can lead to subclavian artery thrombosis with right upper extremity embolization. 9 We hereby describe a rare case, in which the transverse process of C6 vertebra has articulation with the transverse process of C7 that is forming the cervical rib, leading to vascular thoracic outlet syndrome (TOS), causing gangrenous changes in the right upper limb.

## Case Presentation

A 65-year-old lady presented with complaints of blackening of third, fourth, and fifth fingers and gangrenous skin patch over the dorsum of right hand for the last 1 week, associated with tingling and numbness over the right hand and fingers. There was history of mild pain over the right hand that was intermittent and more marked when the patient performed manual work. Pain was occasionally associated with cold sensation of the right upper extremity. There was no previous history of such episodes in the past. There was no history of upper limb swelling or Raynaud's phenomenon in cold weather. The patient was a nonsmoker and a nonalcoholic. She was a diabetic on oral hypoglycemic agents. She was previously operated elsewhere for modified radical mastectomy malignancy on the right side 5 years ago, followed by 6 cycles of chemotherapy with no prior history of radiation therapy postmastectomy. No significant family history could be found.

On clinical examination, vitals were normal with no palpable pulsations in right radial and ulnar arteries; however, the brachial artery was palpable. No abnormal findings were noted in the rest of the arterial system and all the remaining pulsations were palpable. There was no suffusion of face or any dilated and engorged neck veins. Capillary refill was delayed in the right hand. Physical examination did not show evidence of any locally tender areas or neck masses. There was no evidence of muscular atrophy of the right upper extremity; however, there was mottling of skin over palmar aspect of ring finger and dry gangrenous changes on tips of third, fourth, and fifth digits. A complete neurological examination did not reveal any local motors or sensory deficits.

Plain radiograph of the chest showed a cervical rib on the right side. Computed tomography scan (CT) of the neck confirmed right-sided cervical rib arising from the transverse process of C7 vertebra with fusion of the transverse process of C6 vertebrae that was projecting down. Right upper limb arterial Doppler showed an acute to subacute complete lumen occluding thrombus in the right ulnar artery, as well as in the right distal radial artery with good collateral to maintain distal flow. CT angiography of the right upper limb was suggestive of a focal thrombosis of the subclavian artery at the thoracic outlet in costoclavicular space due to extrinsic compression by the cervical rib. There was no evidence of any aneurysm or poststenotic dilatation.

**Fig. 1:**
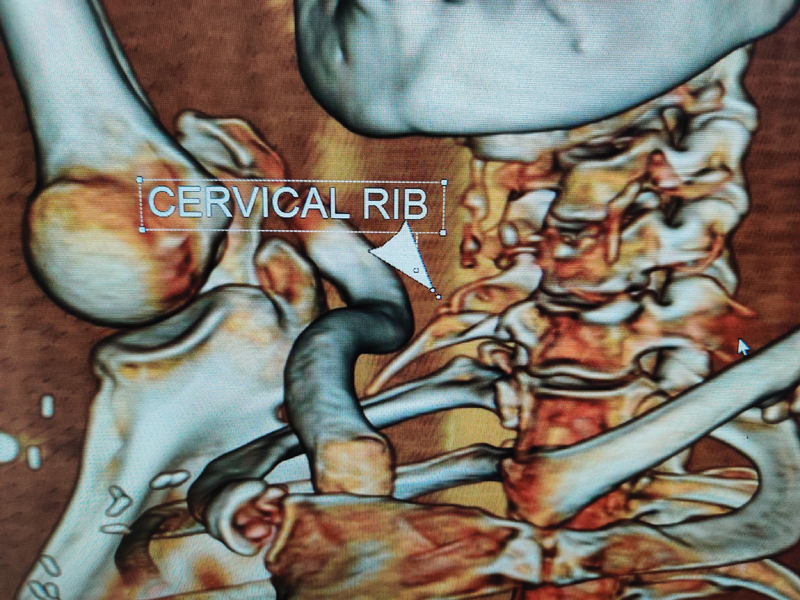
Three-dimensional virtual reconstruction images show fusion of tip of right transverse process of sixth cervical vertebra with the cervical rib.

Gangrene of the lateral fingers with low grade infection warranted local amputation; however, without treating the etiology, the expected result would have been unfavorable. Hence, initially patient was treated with antibiotics with analgesics. The patient received anticoagulation as per our institutional protocol. Patient was initially started on heparin for which was overlapped with warfarin for 5 days, followed by discontinuation of heparin after day 5. Patient was initially started on heparin which was overlapped with warfarin for 5 days, followed by discontinuation of heparin after 5 days. After optimization, she was operated for excision of the cervical rib through the supraclavicular approach ( Fig. 1 ).

### Surgery

**Fig. 2:**
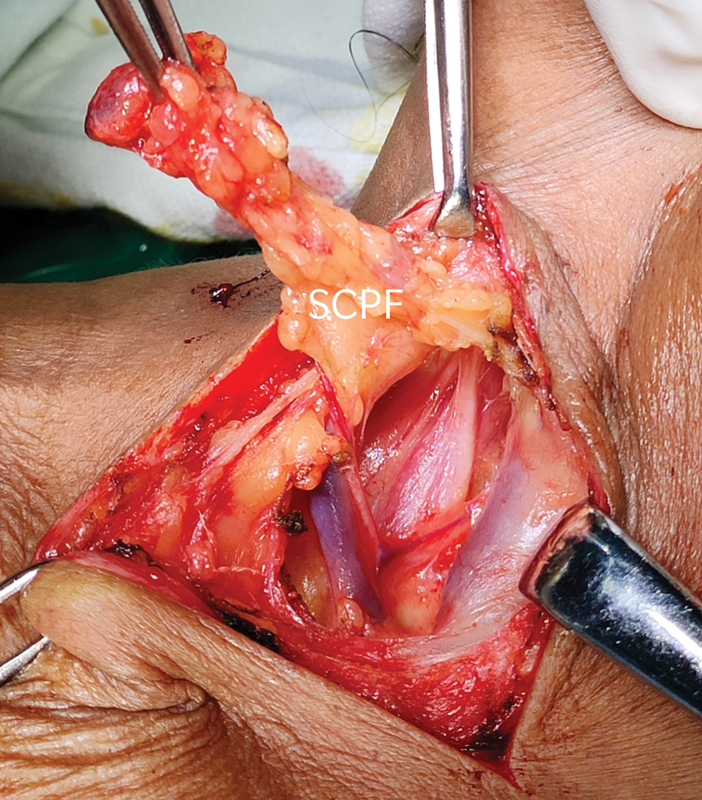
Supraclavicular/scalene pad of fat retracted posteriorly.

**Fig. 3:**
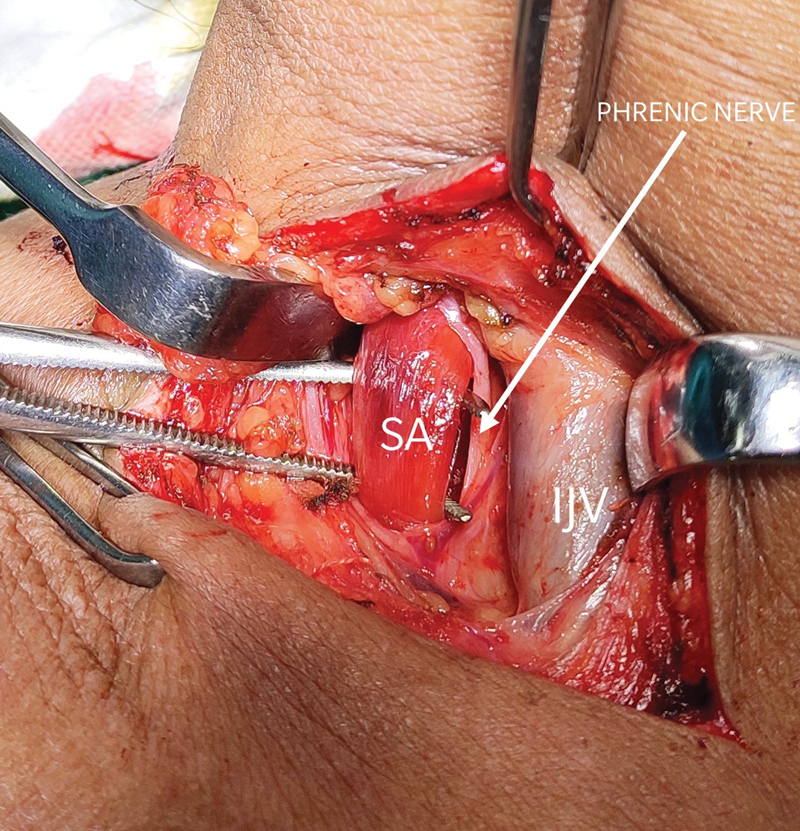
Internal jugular vein identified and retracted medially, phrenic nerve dissected away medially, and scalenus anticus hooked using Mixter forceps.

**Fig. 4:**
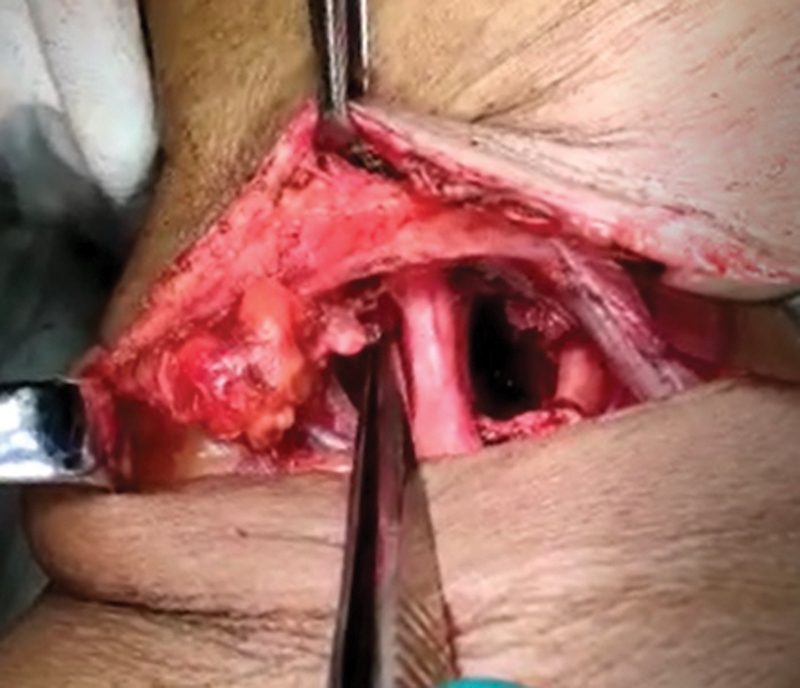
Scalenus anticus cut at the insertion and brachial plexus neurolyzed.

- A supraclavicular exploration was planned for cervical rib excision.
- Under general anesthesia, the patient was positioned supine with an underlying shoulder bump for neck extension and head end was elevated to 30 degrees.
- With the neck extended and turned to left side, a transverse skin incision was taken 2 cm above and parallel to the clavicle.
- Subplatysmal flaps were created and sternocleidomastoid muscle was retracted medially to identify anterior scalene fat pad. Fat pad opened and retracted laterally; inferior belly of omohyoid was identified and cut to visualize the thoracic outlet ( Fig. 2 ).
- Phrenic nerve was identified and retracted medially along with the internal jugular vein to expose the belly and tendon of scalenus anticus.
- Scalenus anticus was cut near its insertion after lifting with Mixter forceps and subclavian artery was identified.
- Neurolysis of the brachial plexus was performed to separate the cords from scalenus medius on the lateral side.
- Long thoracic nerve of bell was identified on posterolateral aspect of scalenus medius and secured by lateral retraction. Scalenus medius was cut near the first rib.
- The cervical rib along with the long transverse process of the sixth vertebra was identified by palpation and osteotomy was performed using hammer and chisel. Bone fragments were excised followed by hemostasis.
- Hemostasis was checked, and wound was closed with suction drain in situ. Ray's amputation of the right fourth finger along with debridement of the gangrenous skin on the right hand ( Figs. 3 and 4).

### Postoperative Course

**Fig. 5:**
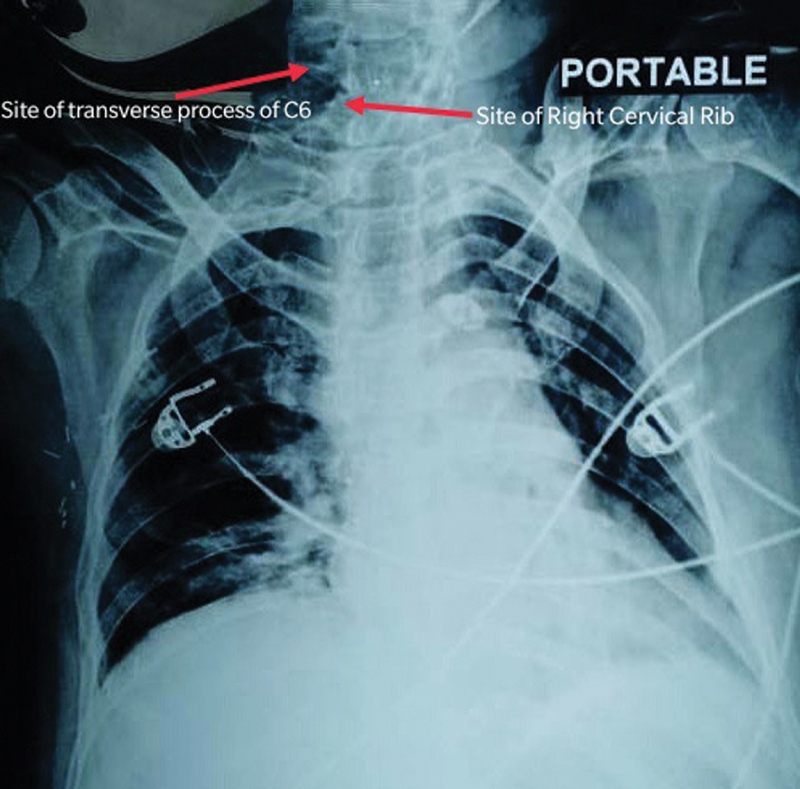
Postoperative chest X-ray shows that the right cervical rib and transverse process of sixth vertebra have been excised.

Patient was started on orals after 6 hours and mobilized. There was no weakness, loss of sensation, or winging of scapula on the right side. She was maintained on oral anticoagulation for 6 weeks and discharged on day 3 of the surgery. Arterial Doppler done after a week and 6 weeks showed radial artery biphasic pattern. Patient was continued on oral anticoagulation and was discharged on warfarin after ensuring the international normalized ratio is in therapeutic range. The wound on the hand healed with no progression of the gangrene at 3 months follow-up. At follow-up of 6 months, there was complete recanalization of the subclavian artery lumen on arterial Doppler ( Figs. 5 6 7 ).

**Fig. 6:**
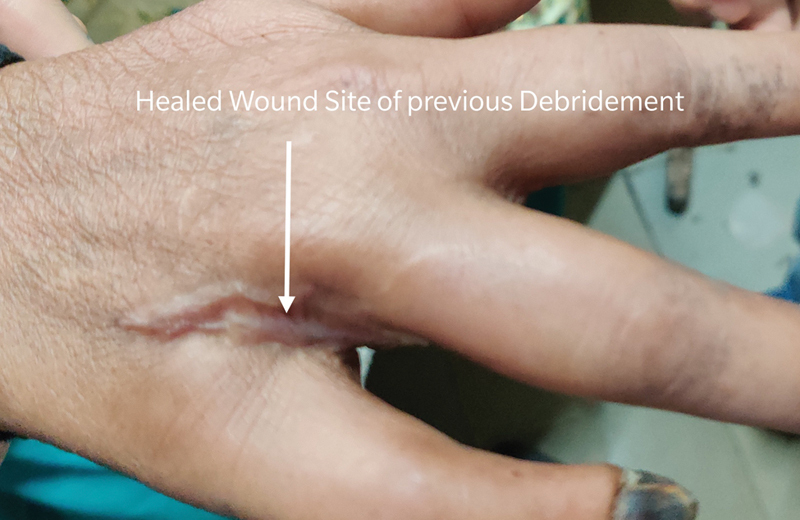
Healed wound at the site of previous debridement on dorsum of right hand at 3 months follow-up.

**Fig. 7:**
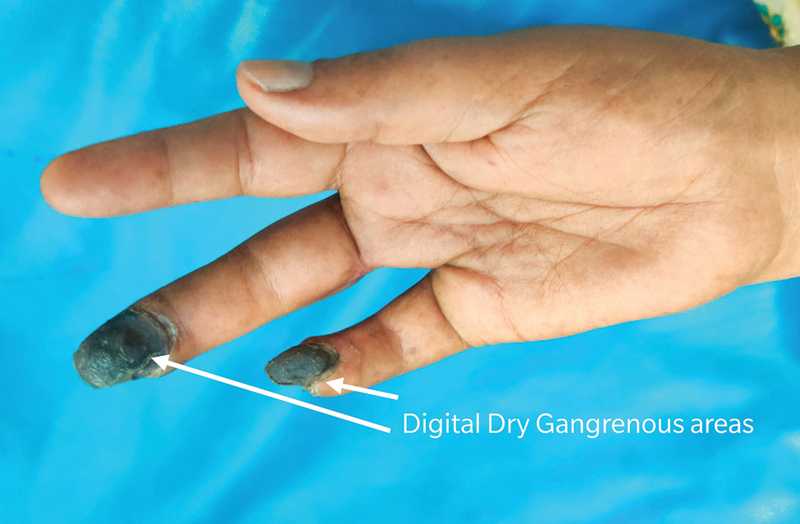
No progression of digital dry gangrene at 3 months follow -up.

## Discussion

A cervical rib extends laterally and forward usually from the transverse process of C7, into the posterior triangle of the neck, where it may terminate as a free end or may join the first thoracic rib, via a fibrous band. It varies in shape, size, direction, and mobility. If it reaches far enough forward, part of the brachial plexus and the subclavian artery and vein cross over it and are apt to sustain compression, thus leading to the clinical symptomatology.

Elongation of the anterior tubercle of the transverse process of the sixth cervical vertebra is a rare finding that was first described by Lapayowker in 1960. 4 Developmentally, the anterior segment of the costal portion of the transverse process may develop from a separate center that appears on the cartilage in the sixth fetal month, fusing with the main ossification center of the transverse process by the sixth year. The anterior tubercle of the transverse processes of the cervical ribs is the homologue of the thoracic ribs, and elongation of the anterior tubercle of a cervical vertebral transverse process suggests another manifestation of a cervical vertebral costal component and is similar to a cervical rib. Aberrant articulation between the two anterior tubercles, as is fusion of the fifth and sixth cervical vertebrae in this case, has only been reported twice in the literature. 7 8 It is a similar finding that occurs at the lumbosacral junction between the enlarged transverse process of a transitional fifth lumbar vertebra, and the ala of the sacrum. 10 Accessory ribs are permanent structures as opposed to ossification sites that disappear postnatally, probably becoming part of the lateral transverse vertebral processes. 2 Ribs are normally present in the fetus in articulation with vertebrae above the eighth, and after birth they are present only as transverse processes of the cervical vertebrae. The extent of growth of a cervical rib is determined by the resistance of the nerve in its path. 11 Vascular symptoms result from irritation of sympathetic fibers in the lowest trunk of brachial plexus. The distal parts of costal processes in seventh cervical vertebra that do not develop normally occasionally develop into cervical a rib. 12

The cervical rib is common in females than males, although not significantly. In case of symptomatic individuals, females more commonly presented with TOS. 13 The clinical manifestations of neurovascular compression caused by cervical ribs were first observed by Cooper in 1818. 14

Depending upon the ossification, cervical ribs are classified broadly into complete or incomplete types. The complete cervical ribs are known to produce vascular symptoms. The symptoms of a cervical rib may be neurogenic (95%) most commonly followed by venous (4–5%), then arterial (1%). 15 Neurogenic TOS is due to brachial plexus compression, associated with chronic inflammation of the scalene muscles, forming tendinous bands, causing compression. Venous TOS, due to venous impingement, presents with arm swelling, cyanosis, and pain due to subclavian vein obstruction, with or without thrombosis. The patients have either hypercoagulable disorders or they may present with effort thrombosis (Paget–Schroetter syndrome). 16 It requires thrombolysis followed by surgical decompression of the subclavian vein. 17 Arterial TOS is usually due to emboli arising from subclavian artery thrombosis, within the poststenotic dilatation due to compression. Symptoms are those of arterial ischemia, & as in this case may have gangrenous changes. Compression may be due to a cervical rib, an anomalous first rib or due to fibrocartilaginous bands in the thoracic outlet. This may be visible on a regular X-ray of the chest. Prevention of adverse vascular events requires early detection of lesions like subclavian artery compression with poststenotic dilatation or an aneurysm, which cause embolism and are responsible for the same. Symptomatology of TOS may also include pain over the extremity with or without paraesthesia, neck pain, and occipital headache. The possible sites of compression of the neurovascular bundle are the interscalene triangle, costoclavicular space, and retropectoralis minor space. Compression in the interscalene triangle, known as scalene syndrome, has neurological and arterial symptoms; there are no venous symptoms, since the subclavian vein is not contained in this triangle. Costoclavicular compression syndrome can compress any bundle structure. 18

TOS can be managed conservatively or surgically. Conservative line of management includes appropriate physical therapy, bed rest, and avoidance of precipitating factors. Appropriate analgesia is the key in cases with isolated neurogenic TOS. Failure of conservative management, that is, persistent symptoms of neurovascular compression despite 6 months of therapy or symptom recurrence or no improvement in nerve conduction velocities warrants surgical treatment. Cervical rib excision or scalenotomy is sufficient to relieve the symptoms of TOS with or without excision of the first rib. 18

There are multiple surgical approaches for thoracic outlet decompression as transaxillary, supraclavicular, and infraclavicular approaches. 19 20 Depending upon the anatomic abnormalities that are identified and surgeon preference, the approach may be chosen. The transaxillary approach to thoracic outlet decompression has the advantage of more complete visualization of the rib during resection, but it does not allow for vascular reconstruction. The approach requires a limited amount of dissection, and external scarring is minimized. The supraclavicular approach provides a wider exposure of the ribs, and the site of compression can be easily identified. Arterial reconstruction can be performed, as needed. The complete resection of anterior and middle scalene muscles can be performed with this approach, along with brachial plexus neurolysis, as in this case. Resection of the first rib may or may not be required to achieve sufficient decompression.

We preferred the supraclavicular approach for cervical rib excision in our patient anticipating the novel anatomy of this variant preoperatively. The surgical access and the complete excision of the cervical rib would have been difficult via the infraclavicular approach, as it was arising from C6 and the C7 vertebra. Consideration was also given to the risk of subclavian arterial injury intraoperatively and the requirement for vascular reconstruction that would have been difficult with the transaxillary approach.

Occasionally, an infraclavicular incision is needed to fully see the venous structures. The infraclavicular approach is particularly useful in cases of vascular TOS that require extensive venous reconstruction. Exposure of the central veins can be achieved by extending the incision medially and performing a transverse sternotomy. Complications of the surgery include phrenic nerve injury, subclavian artery, and vein lesions. Pneumothorax is rare if pleura is damaged inadvertently.

A collaborative interdisciplinary approach, among the various medical specialities, is the key to successfully treat TOS, regardless of the type of symptoms.

## Conclusion

A high index of suspicion is required for considering cervical rib as potential cause for arterial TOS. A surgical intervention is strongly recommended in cases of symptomatic cervical ribs, presenting with arterial TOS that may decrease the morbidity of the patient.
